# Author’s Reply: The Role of Visceral Obesity, Sarcopenia and Sarcopenic Obesity on Surgical Outcomes After Liver Resections for Colorectal Metastases

**DOI:** 10.1007/s00268-021-06282-2

**Published:** 2021-08-18

**Authors:** Mira Runkel

**Affiliations:** Department of General and Visceral Surgery, Medical Center- University of Freiburg, Hugstetterstrasse 55, 79106 Freiburg, Germany

We appreciate the interest taken by Furukawa et al*.* in our article ‘The role of visceral obesity, sarcopenia and sarcopenic obesity on surgical outcomes after liver resections for colorectal metastases’ [1]. We thank you for the stimulating comment about the complexity and importance of body composition in oncological surgery.

Osteoporosis and osteopenia have always been part of the process of ‘normal’ aging and play a crucial role in the frailty syndrome. Up until now, these body compositions have been investigated as separate entities; however, it is suggested that the combination of extremes in body compositions, including sarcopenia, obesity and osteoporosis, could lead to higher morbidity, mortality and decrease in functionality [2, 3].

The quantitative loss of bone and loss of bone density (osteopenia/osteoporosis) and the loss of muscle mass and function (sarcopenia) share similar risk factors, including genetics, mechanical and endocrine factors [3]. Additionally, the redistribution of fat is not only seen between subcutaneous and visceral fat areas, but adipogenesis is also seen within bone and muscle mass, which could further reduce functionality. Clearly, these entities are linked in their presentation as well as in the underlying pathology, and investigation and treatment should focus on the combination of body compositions rather than on the separate entities [2, 4]. The ‘triple’ burden of osteosarcopenic obesity has been originally described in 2014, and although the understanding of this complex disease is limited, it is assumed that patients with osteosarcopenic obesity suffer from poorer clinical outcomes, including high risk of fractures, impaired functional status, physical disability, insulin resistance, increased risk of infection, increased length of hospital stay and reduced overall survival [4, 5]

Evidence is limited as only few reviews and retrospective studies exist that concern diagnosis, prevalence and the clinical relevance of osteosarcopenic obesity [2–7]. Illich et al*.* investigated 258 postmenopausal women and assessed functionality with regard to different body compositions. Patients with osteosarcopenic obesity had overall worse functionality compared to other groups [8]. Significantly lower physical performance and frailty were shown for osteosarcopenic obese middle and older aged women in a prospective study [9]. Interestingly, the presence of osteosarcopenic obesity was shown in phenotypically healthy obese Greek patients [6] Despite a growing interest in the clinical relevance of osteosarcopenic obesity, there is a lack of larger series or prospective studies.

Similar to the problems discussed in our paper [1], multiple definitions and cut-off points for body compositions are a hurdle for unbiased, prospective research. Fortunately, the definition and cut-off values for osteopenia and osteoporosis are well established and based on DXA scans. DXA scans, however, provide a limitation in availability and are usually not part of routine screening. New methods of non-invasive bone measurement, including bioelectrical impedance, are currently being tested but are not part of routine clinical workup.

Certainly, the idea about investigating the additional effect of osteosarcopenic obesity on surgical outcome and survival is timely and interesting. Although recognized as part of the aging population, its existence might even extend to younger populations [6]. Osteopenia/osteoporosis in combination with sarcopenia and obesity may further increase the risk of postoperative complications and overall survival. Ideally, prospective research should be carried out by adding a DXA scan to assess bone density and using preoperative CT scans for the calculation of visceral fat and lean muscle mass.
